# The miR-4739/DLX3 Axis Modulates Bone Marrow-Derived Mesenchymal Stem Cell (BMSC) Osteogenesis Affecting Osteoporosis Progression

**DOI:** 10.3389/fendo.2021.703167

**Published:** 2021-12-02

**Authors:** Ding Li, Qi Yuan, Liang Xiong, Aoyu Li, Yu Xia

**Affiliations:** ^1^ Department of Orthopedics, The Second Xiangya Hospital, Central South University, Changsha, China; ^2^ Department of Hepatopathy, The Hunan Provincial People’s Hospital, The First Affiliated Hospital of Hunan Normal University, Changsha, China

**Keywords:** Osteoporosis, bone marrow-derived mesenchymal stem cell (BMSC), osteogenic differentiation, miR-4739, DLX3

## Abstract

Osteoporosis is a complex multifactorial disorder linked to various risk factors and medical conditions. Bone marrow-derived mesenchymal stem cell (BMSC) dysfunction potentially plays a critical role in osteoporosis pathogenesis. Herein, the study identified that miR-4739 was upregulated in BMSC cultures harvested from osteoporotic subjects. BMSCs were isolated from normal and osteoporotic bone marrow tissues and identified for their osteogenic differentiation potential. In osteoporotic BMSCs, miR-4739 overexpression significantly inhibited cell viability, osteoblast differentiation, mineralized nodule formation, and heterotopic bone formation, whereas miR-4739 inhibition exerted opposite effects. Through direct binding, miR-4739 inhibited distal-less homeobox 3 (DLX3) expression. In osteoporotic BMSCs, DLX3 knockdown also inhibited BMSC viability and osteogenic differentiation. Moreover, DLX3 knockdown partially attenuated the effects of miR-4739 inhibition upon BMSCs. Altogether, the miR-4739/DLX3 axis modulates the capacity of BMSCs to differentiate into osteoblasts, which potentially plays a role in osteoporosis pathogenesis. The *in vivo* and clinical functions of the miR-4739/DLX3 axis require further investigation.

## Introduction

Osteoporosis is a complex multifactorial disorder that is related to various risk factors and medical conditions. Three common types of osteoporosis are senile osteoporosis, sex steroid deficiency osteoporosis, and secondary osteoporosis (1–5). Moreover, in postmenopausal women with osteoporosis, the aging of osteocytes and their microenvironment, low levels of estrogen, and increased reactive oxygen species (ROS) production provoke a decline in bone formation and an increase in bone resorption (2, 6). Reportedly, bone regeneration through BMSCs infusion could trigger osteogenesis, thereby providing a potential therapeutic strategy for primary osteoporosis (7). The identification of the alterations occurring in the bone marrow population dynamics, namely the dysfunction of BMSCs, is essential for designing optimized strategies to restore bone formation.

MicroRNAs (miRNAs) are small, single-stranded non-coding RNAs having an average of 22 nucleotides in length. miRNAs exert their biological functions through the downregulation of gene expression *via* translational repression or degradation of mRNA targets (8, 9). As per recent reports, multiple miRNAs could potentially play a part in osteogenesis (10–12). Several studies indicated the potential roles of miRNAs in osteoporosis pathogenesis, affecting BMSC osteogenic differentiation (13–16). For instance, Li et al. (13) indicated that miR-188 is an important regulator of the age-associated switch between osteoblast and adipocyte differentiation of BMSCs, representing an underlying therapeutic target for age-associated bone loss. Hu et al. (15) reported that miR-26b modulated osteoactivin-induced osteoblast differentiation of BMSCs *via* the GSK3β/β-catenin signaling. Over the past decades, RNA-sequencing and miRNA-microarray have been performed to identify differentially expressed miRNAs in the BMSCs in control or osteoporotic subjects or at different stages of osteogenic differentiation. For instance, Wang and colleagues analyzed RNA-seq and miRNA-microarray data for differentially expressed between the ovariectomized (OVX) mice and controls, and 22 miRNAs were identified (17). Considering that miRNAs exert regulatory roles in target gene expression, identifying more miRNA/mRNA axes modulating BMSC osteogenic differentiation could potentially provide potential agents for osteoporosis treatment.

In this study, online microarray chip data from Gene Expression Omnibus (GEO) were analyzed to select miRNAs differentially expressed in osteoporotic subjects, and miR-4739 was subsequently selected. BMSCs were isolated from normal and osteoporotic bone marrow tissues, respectively, and identified. BMSCs were induced towards osteogenic differentiation, and the differentiation was verified. miR-4739 overexpression or inhibition was achieved in BMSCs, and the effects of miR-4739 upon osteogenesis were then examined. Downstream targets of miR-4739 were analyzed, and DLX3 was selected. Predicted miR-4739 binding to DLX3 and miR-4739 regulation of DLX3 were investigated. The co-effects of the miR-4739/DLX3 axis upon BMSC osteogenesis were determined.

## Materials And Methods

### Tissue Sample Collection

Osteoporotic bone marrow tissues were harvested from patients (n=5, 62.80 ± 6.61 years, female/male is 4/1) who were diagnosed with osteoporosis and who underwent hip surgery. The normal bone marrow tissues were donated by the patients who underwent lower limbs traumatic fracture (n=5, 53.20 ± 4.32 years, female/male is 3/2). No differences were observed in age, BMI, serum vitamin D status, serum calcium, and parathyroid hormone status between the controls and osteoporosis patients. All tissue samples were collected under sterile conditions and transferred to a tube containing heparin anticoagulant immediately after resection. Informed consent was signed by each enrolled patient, and the sampling was approved by the Ethics Committee of the Second Xiangya Hospital of Central South University [approval No. 2018(Yan001)].

### Isolating BMSCs From Bone Marrow Tissues

BMSCs were isolated from osteoporotic or normal bone marrow tissues following the aforementioned methods (18, 19). Collected BMSCs were maintained in Dulbecco modified Eagle’s medium (DMEM; Invitrogen, Carlsbad, CA, USA) supplemented with 10% heat‐inactivated FBS and 1% penicillin, at 37°C in 5% CO_2_. The medium was replaced with fresh medium every 2 days. Passage 2-5 BMSCs were used in all experiments. Flow cytometry was performed to identify isolated osteoporotic and normal BMSCs by detecting CD34, CD45, CD73, CD90, and CD105.

### Flow Cytometry

When the density of the BMSCs reached 80%, BMSCs were trypsinized, centrifuged, and the supernatant was discarded. Cells were resuspended and adjusted to the density of 3-6 × 10^3^ cells/μl. CD73, CD90, CD105, CD45, and CD34 antibodies (obtained from Abcam, Cambridge, MA, USA) were used for a 30-min incubation at room temperature for cell labeling, using untreated BMSCs and isotype-control (FITC- or PE-labeled Mouse IgG1 obtained from Abcam) as controls.

### Bioinformatics Analysis

To identify miRNAs or genes potentially regulating the osteogenic differentiation during osteoporosis, the Gene Expression Omnibus (GEO) datasets, including GSE74209 (Specific miRNAs profiles in Fresh femoral neck trabecular bone from 12 postmenopausal women who had undergone hip replacement due to either osteoporotic fracture or osteoarthritis without osteoporosis) (20), GSE93883 (Specific miRNAs profiles in the plasma from non-osteoporotic patients and osteoporotic patients with and without vertebral fractures), and GSE80614 (different genes expression profile in human BMSC and differentiation osteoblast) (21) were downloaded using the R language GEOquery package. The differential expression miRNAs or genes were analyzed by the Limma package. For the selection of miR-4739 targeted genes, miRDIP (22) (https://ophid.utoronto.ca/mirDIP/) was used to predict the miR-4739 targeted genes.

### Real-Time Reverse Transcription-Polymerase Chain Reaction (qRT-PCR) Analysis

The TRIzol reagent (Invitrogen, Carlsbad, CA, USA) was used for extracting total RNA from target cells. A PrimeScript RT kit (Takara Bio, Shiga, Japan) was used for total RNA reverse transcription. An ABI Prism 7500 Sequence Detection System (Applied Biosystems, Foster City, CA, USA) was used to run the reaction using the SYBR Green Quantitative Kit (Toyobo, Osaka, Japan). The level of U6 (for miRNA) or GAPDH (for mRNA) was used as an internal reference. The relative expression levels of each factor were calculated using the 2^ΔΔ−ct^ method. The primers are listed in **Table S1**.

### Protein Isolation and Immunoblotting

The RIPA buffer containing protease inhibitors (Beyotime, Shanghai, China) was used to extract protein samples from target cells. The BCA quantitative method (Beyotime) was used to quantify the protein sample concentration. SDS-PAGE (10%; Invitrogen) was used to separate collected protein samples, which were then transferred to PVDF membranes (Millipore, Burlington, MA, USA). BSA (5%) was used to incubate the membranes for 2 h to prevent non-specific bindings. The following antibodies were used to incubate the membranes at 4°C overnight: ALP (ab67228, Abcam), OCN (ab93876, Abcam), RUNX2 (ab76956, Abcam), Osterix (ab209484, Abcam), DLX3 (ab178428, Abcam). Proper secondary antibodies (Cowin Biotech Co, Beijing, China) were used to incubate the membranes at room temperature for 2 h after the primary antibody incubation. Enhanced Chemiluminescence (ECL) Fluorescence Detection Kit (BB-3501; Amersham Pharmacia, Piscataway, NJ, USA) was used to visualize the blot signal on a Bio-Rad image analysis system (Bio-Rad, Hercules, CA, USA). The relative protein content is expressed by the gray value of the corresponding protein band/GAPDH protein band.

### Alkaline Phosphatase (ALP) Staining

BMSCs were cultured in an osteogenic induction culture medium (complete culture medium contains 5 mM β-glycerophosphate, 50 μM ascorbate, and 100 nM dexamethasone) for 14 days and were stained with the ALP staining solution (Nanjing Jiancheng, Nanjing, China) on day 0, 7, and 14 of osteogenic induction. The staining results were observed under a microscope (Olympus, Tokyo, Japan), and representative images were photographed.

### Alizarin Red Staining

BMSCs were cultured in an osteogenic induction culture medium for 21 days, and alizarin red staining was performed on days 0 and 21 of osteogenic induction as previously described (23). In general, the differentiated BMSCs were washed twice with PBS, fixed in isopropanol (60%) for 1 min at room temperature, washed twice with ddH_2_O, and dyed with 1% Alizarin Red (Sigma-Aldrich, St. Louis, USA, MO) for 10 min at room temperature. A microscope (Olympus) was used to observe the staining and representative images were photographed.

### Cell Transfection

miR-4739 overexpression or inhibition was achieved by transfecting agomir-4739 or antagomir-4739 (GenePharma, Shanghai, China). DLX3 knockdown was achieved by transfecting the shRNA vector containing short hairpin RNA against DLX3 (sh-DLX3, GenePharma). The transfection was performed using Lipofectamine 3000 (Life Technologies, Carlsbad, CA, USA) as per the aforementioned methods (24); 48 h after transfection, cells were harvested for subsequent experiments.

### Cell Counting Kit 8 (CCK-8) Assay for Cell Viability

Cells, either treated or transfected, were seeded into 96-well plates at a density of 2 × 10^3^ cells per well and incubated with 10 μl CCK-8 solution (Dojindo Co. Tokyo, Japan) at 37°C for 3 h. A microplate reader was then used to determine the optical density (OD) value at the wavelength of 450 nm at the end of the incubation with CCK-8 solution.

### Dual-Luciferase Reporter Assay

Wild- and mutant-type DLX3 3’UTR luciferase reporter vectors were constructed based on psiCheck-2 (Promega, Madison, WI, USA) and named wt-/mut-DLX3; mut-DLX3 contains a 5-bp mutation in the predicted miR-4739 binding site. Reporter vectors were co-transfected in 293T cells with agomir-4739/antagomir-4739, and the luciferase activity was subsequently determined.

### Heterotopic Bone Formation Assay *In Vivo*

BMSCs were transfected with miR-4739 antagomir mimics and mimics-NC for 24 h. Hydroxyapatite (HA) powder (40 mg, Sigma, USA) was diluted with 200 μl of the standard growth medium and mixed with BMSCs (5×10^6^ cells) and incubated at 37°C for 2 h in 5% CO_2_. Next, the BMSCs-HA mixtures were injected subcutaneously on the dorsal side of 6 weeks old BALB/C nude mice. Six weeks later, the mice were sacrificed, and the implants were collected. All the animal experiments were approved by the Animal Care and Use Ethics Committee of Central South University [approval No. 2018(Yan001)].

### Histological Analysis

The heterotopic bone tissues were fixed in 10% buffered formalin for 48 h, decalcified using an EDTA-Decalcifying-fluid (Boster, Wuhan, China). The decalcified bone tissues were then embedded in paraffin. Histological analyses were performed on specimen cross-sections 5-μm thick stained with hematoxylin-eosin (HE) and Masson staining using the HE stain kit (Boster) and Masson’s trichrome stain kit (Solarbio, Beijing, China). The sections were observed through an optical microscope (Olympus). The semiquantitative image analysis was performed by ImageJ (NIH, USA).

### Statistics Analyses

Statistical analyses were carried out using GraphPad software. Data were reported as mean ± SD based on at least three replicates. *P* values are shown in the figures or figure legends. *P* values were calculated with unpaired Student’s *t*-test for two groups or analysis of variation (ANOVA) with Tukey’s *post-hoc* for experiments with more than two groups. *P* values less than 0.05 were considered statistically significant.

## Results

### miR-4739 Expression Is Upregulated in Osteoporotic BMSCs

**Figure 1:**
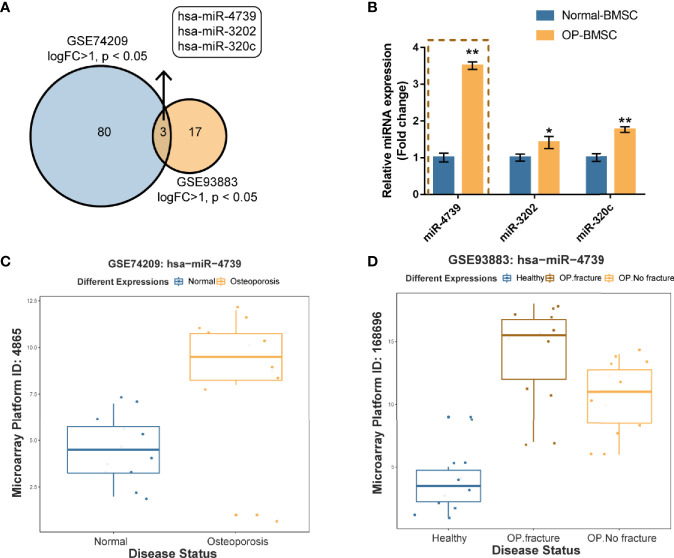
miR-4739 expression is deregulated in osteoporotic bone marrow-derived mesenchymal stem cells (BMSCs) **(A)** miRNAs differentially expressed in postmenopausal women undergoing hip replacement due to either osteoporotic fracture or osteoarthritis in the absence of osteoporosis (GSE74209) or plasma from non-osteoporotic and osteoporotic patients with and without vertebral fractures (GSE93883) were compared (logFC >1, p < 0.05) and 3 miRNAs (miR-4739, miR-3202, and miR320c) were overlapped in two datasets. **(B)** The expression of miR-4739, miR-3202, and miR-320c in normal and osteoporotic BMSCs was determined by RT-PCR. (n=3) **(C, D)** The expression of miR-4739 is based on GSE74209 and GSE93883. *p < 0.05, **p < 0.01.

To identify miRNAs that potentially regulate the osteogenic differentiation of osteoporotic BMSCs, differentially expressed miRNAs were analyzed using online microarray chip data from Gene Expression Omnibus (GEO) (**Figure 1A**). According to GSE74209 containing miRNAs differentially expressed in postmenopausal women experiencing hip arthroplasty due to either osteoporotic fracture or osteoarthritis without osteoporosis (**Figure 1C**, **Figure S1A** and **Table S2**) and GSE93883 containing miRNAs differentially expressed in non-osteoporotic patients and patients with osteoporosis in the presence or absence of vertebral fractures (**Figure 1D**, **Figure S1B** and **Table S2**), 3 upregulated miRNAs (miR-4739, miR-3202, and miR320c) were overlapped in two datasets (**Figures 1A, C, D**). In osteoporotic BMSCs, miR-4739 expression was mostly upregulated when compared with normal BMSCs (**Figure 1B**). Thus, miR-4739 was selected for subsequent experiments.

### Isolation and Osteogenic Differentiation of BMSCs

**Figure 2:**
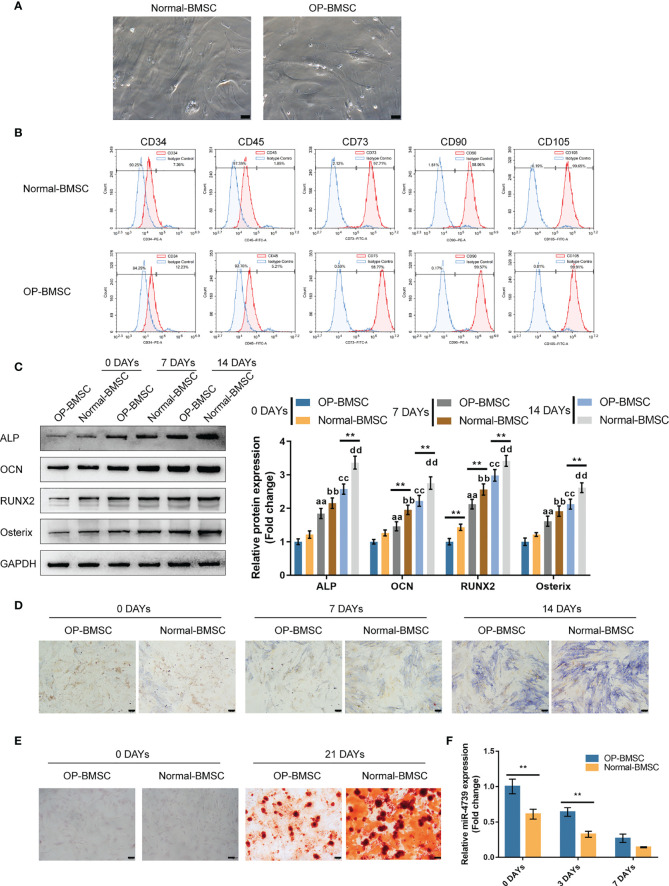
Isolation and osteogenic differentiation of BMSCs **(A)** Normal and osteoporotic BMSCs were isolated from normal and osteoporotic bone marrow tissues, respectively (the scale bar is 50 μm). **(B)** Flow cytometry was performed to identify isolated BMSCs detecting CD34, CD45, CD73, CD90, and CD105. **(C)** Normal and osteoporotic BMSCs were induced towards osteogenic differentiation for 21 days, and the protein levels of ALP, OCN, Runx2, and Osterix were examined using Immunoblotting on days 0, 7, and 14 of osteogenic induction **(C)**; ALP staining was performed on day 0, 7, and 14 of osteogenic induction **(D)**; the formation of mineralized nodules were examined using Alizarin red staining on days 0 and 21 of osteogenic induction **(E)**; the expression of miR-4739 was examined using qRT-PCR on day 0, 3, and 7 of osteogenic induction in normal BMSCs and osteoporotic BMSCs **(F)**. n=3, **p < 0.01 compared to OP-BMSC. aa p<0.01 compared between 0 days OP-BMSC and 3 days OP-BMSC; bb p < 0.01 compared between 0 days normal-BMSC and 3 days normal-BMSC; cc p<0.01 compared between 0 days OP-BMSC and 14 days OP-BMSC; dd p < 0.01 compared between 0 days normal-BMSC and 14 days normal-BMSC.

To investigate the effect of miR-4739 on osteoporotic BMSC osteogenesis, the study isolated normal and osteoporotic BMSCs from normal and osteoporotic bone marrow tissues, respectively (**Figure 2A**). The identification of BMSCs was conducted using Flow cytometry detecting CD34, CD45, CD73, CD90, and CD105; as illustrated in **Figure 2B**, BMSCs were CD34-negative, CD45-negative, CD73-positive, CD90-positive, and CD105-positive. Normal and osteoporotic BMSCs were induced towards osteogenic differentiation for 21 days and examined for differentiation. On days 0, 7, and 14, the protein levels of ALP, OCN, Runx2, and Osterix were examined. As depicted in **Figure 2C**, the levels of all the four markers increased time-dependently; on day 14, the levels of the four markers in normal BMSCs exceeded those in osteoporotic BMSCs, suggesting the impaired osteogenic differentiation potential of osteoporotic BMSCs. Consistently, ALP staining confirmed the osteoblast differentiation in both types of BMSCs on days 7 and 14, and Alizarin red staining confirmed the formation of mineralized nodules in both types of BMSCs on day 21; the osteoblast differentiation and mineralized nodule formation ability in osteoporotic BMSCs were impaired (**Figures 2D, E**). Meanwhile, miR-4739 expressions showed to be downregulated in the process of osteogenic differentiation time-dependently (**Figure 2F**).

### Effects of miR-4739 Overexpression and Inhibition on Osteoporotic and Normal BMSC Osteogenic Differentiation

**Figure 3:**
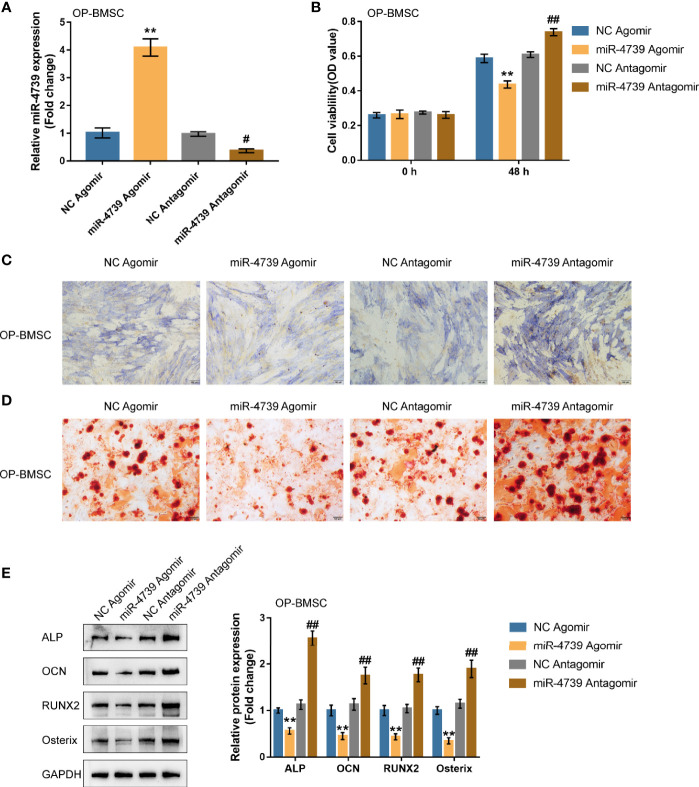
Effects of miR-4739 overexpression and inhibition on osteoporotic BMSC osteogenic differentiation **(A)** miR-4739 overexpression or inhibition was achieved in osteoporotic BMSCs by transfecting agomir-4739 or antagomir-4739; miR-4739 expression was confirmed using qRT-PCR. n=3. **(B)** Osteoporotic BMSCs were transfected with agomir-4739 or antagomir-4739 and examined for cell viability by CCK-8 assay (n=3). Then, osteoporotic BMSCs were transfected with agomir-4739 or antagomir-4739, induced towards osteogenic differentiation for 21 days, and examined using ALP staining on day 14 of osteogenic induction **(C)**; examined for the formation of mineralized nodules using Alizarin red staining on day 21 of osteogenic induction **(D)**; examined for the protein levels of ALP, OCN, Runx2, and Osterix using Immunoblotting on day 14 of osteogenic induction **(E)** (n=3). **P < 0.01, compared to NC agomir; ^#^ *P < *0.05, ^##^ *P < *0.01, compared to NC antagomir.

Considering the downregulation of miR-4739 during BMSC osteogenesis, the specific effect of miR-4739 on BMSC osteogenesis was further validated. The study achieved miR-4739 overexpression or inhibition in osteoporotic BMSCs through the transfection of agomir-4739 or antagomir-4739; qRT-PCR was performed to confirm miR-4739 expression (**Figure 3A**). In osteoporotic BMSCs, miR-4739 overexpression inhibited BMSC cell viability, whereas miR-4739 inhibition exerted opposite effects (**Figure 3B**). Osteoporotic BMSCs were subsequently transfected with agomir-4739 or antagomir-4739, induced towards osteogenic differentiation for 21 days, and examined for the effects of miR-4739 on BMSC osteogenesis. ALP staining showed that, on day 14 of osteogenic induction, miR-4739 overexpression inhibited, whereas miR-4739 inhibition promoted osteoblast differentiation (**Figure 3C**). Alizarin red staining revealed that, on day 21 of osteogenic induction, miR-4739 overexpression inhibited, whereas miR-4739 inhibition promoted the formation of mineralized nodules (**Figure 3D**). Meanwhile, the levels of osteogenic differentiation markers were monitored. On day 14 of osteogenic induction, miR-4739 overexpression decreased, whereas miR-4739 inhibition increased the protein levels of ALP, OCN, Runx2, and Osterix (**Figure 3E**). Moreover, in normal BMSCs, miR-4739 overexpression also inhibited BMSC cell viability, osteogenic ability, and the expression of ALP, OCN, RUNX2, and Osterix proteins, while miR-4739 inhibition exerted opposite effects (**Figure S2**). These data indicate that miR-4739 overexpression impairs the osteogenic differentiation potential of both normal and osteoporotic BMSCs.

**Figure 4:**
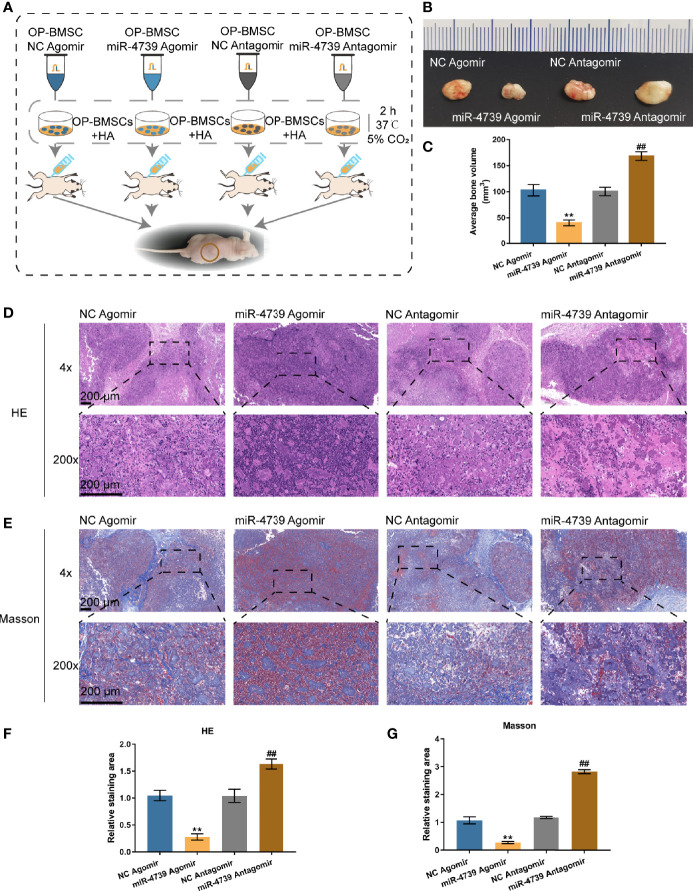
miR-4739 modulated heterotopic bone formation from osteoporotic BMSCs *in vivo*. **(A)** The protocol of heterotopic bone formation. **(B, C)** Gross view and volume of heterotopic bone from miR-4739 agomir or antagomir transfected osteoporotic BMSCs. **(D, E)** Histological analysis of heterotopic bone formation with HE and Masson staining (the scale bar is 200 μm). The relative staining area of HE and Masson was shown in **(F, G)**. n=4. **P < 0.01, compared to NC agomir; ^##^ *P <* 0.01, compared to NC antagomir.

The effects of miR-4739 on bone formation *in vivo* were subsequently determined. MiR-4739 agomir or antagomir-transfected osteoporotic BMSCs were mixed with the osteoconductive carrier HA and injected subcutaneously in nude mice (**Figure 4A**). Six weeks later, the implants were harvested and measured. The results show that miR-4739 agomir transfected osteoporotic BMSCs formed smaller bone masses than NC agomir transfected osteoporotic BMSCs, while miR-4739 antagomir transfected BMSCs formed larger bone masses than NC antagomir transfected BMSCs (**Figures 4B, C**). The heterotropic bone sections were treated with HE or Masson staining. The results revealed that bone areas were significantly decreased in the miR-4739 agomir group and increased in the miR-4739 antagomir, compared to the NC group (**Figures 4D–G**). This result was consistent with the *in vitro* result that miR-4739 inhibited BMSC growth and osteogenic differentiation.

### miR-4739 Targets DLX3 and Inhibits DLX3

**Figure 5:**
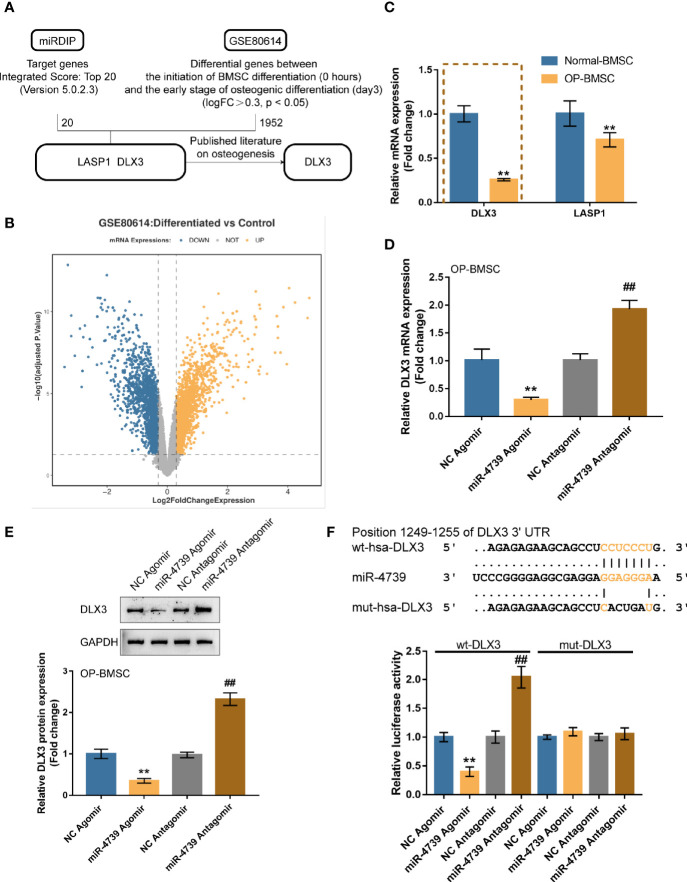
miR-4739 targets DLX3 and inhibits DLX3 **(A, B)** miRDIP was used to predict miR-4739 targets (top 20 highest integrated score genes); differentially expressed genes in BMSCs on day 0 and 3 of osteogenic differentiation were analyzed according to GSE80614 (logFC >0.3, *P <*0.05). These two datasets intersected in LASP1and DLX3. **(C)** The expression of DLX3 and LASP1 in normal or osteoporotic BMSCs. n=3. **(D)** Osteoporotic BMSCs were transfected with agomir-4739 or antagomir-4739 and examined for DLX3 expression using qRT-PCR. n=3. **(E)** DLX3 protein levels using Immunoblotting. n=3. **(F)** Wild- and mutant-type DLX3 luciferase reporter vectors were generated and co-transfected in 293T cells with agomir-4739 or antagomir-4739; the luciferase activity was determined. n=3. **P < 0.01, compared to NC agomir; ^##^ *P < *0.01, compared to NC antagomir.

miRNAs exert their biological functions by targeting the 3’UTR of downstream mRNA (25). Firstly, genes that are potentially targeted by miR-4739 were identified using the online tool miRDIP (https://ophid.utoronto.ca/mirDIP/) to predict the targeted genes. The top 20 highest integrated score genes were selected. The differentially expressed genes in BMSCs on days 0 and 3 of osteogenic differentiation were subsequently selected according to GSE80614 (logFC>0.3, p<0.05). These two datasets intersected in LASP1 (LIM and SH3 Protein 1) and DLX3 (**Figures 5A, B** and **Table S2**). Among them, only DLX3 has been reported to be associated with osteogenesis (26). The expression of DLX3 and LASP1 was subsequently determined in normal BMSCs and osteoporotic BMSCs. As illustrated in **Figure 5C** showed, both DLX3 and LASP1 were downregulated in osteoporotic BMSCs. The reduced fold change of DLX3 is larger than LASP1; consequently, DLX3 was chosen for further investigation (**Figure 5C**). In osteoporotic BMSCs, miR-4739 overexpression downregulated DLX3 mRNA expression and decreased DLX3 protein levels, whereas miR-4739 inhibition exerted opposite effects of DLX3 (**Figures 5D, E**). A dual-luciferase reporter assay was subsequently conducted to confirm the predicted binding between miR-4739 and DLX3 3’UTR. Wild- and mutant-type DLX3 3’UTR luciferase reporter vectors were generated and were co-transfected in 293T cells with agomir-4739/antagomir-4739. The luciferase activity was subsequently examined. When co-transfected with wt-DLX3, miR-4739 overexpression inhibited, whereas miR-4739 inhibition enhanced the luciferase activity; when co-transfected with mut-DLX3, miR-4739 caused no changes in the luciferase activity (**Figure 5F**). These data indicate that miR-4739 targets DLX3 and inhibits DLX3 expression.

### Co-Effects of the miR-4739/DLX3 Axis on Osteoporotic BMSC Osteogenic Differentiation

**Figure 6:**
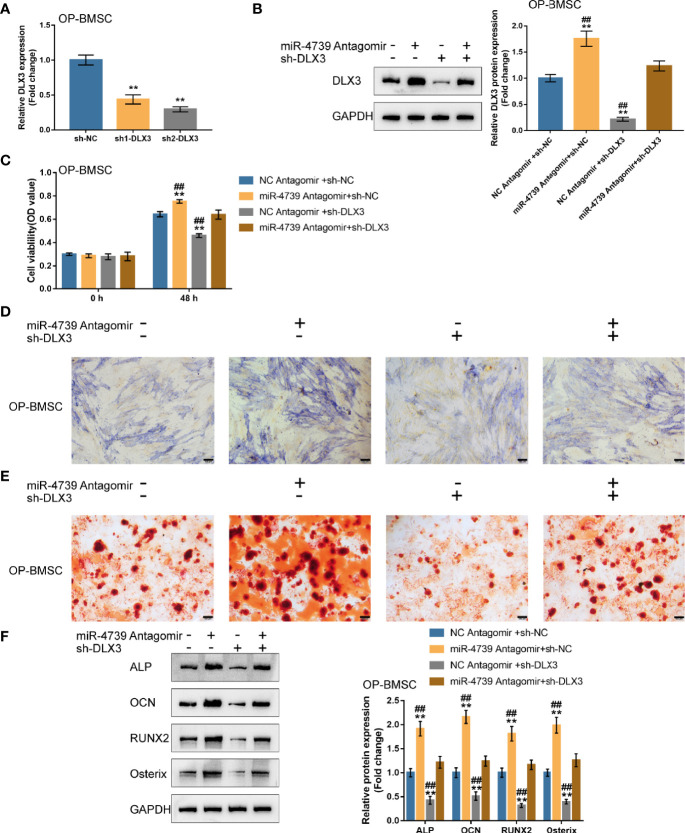
Co-effects of the miR-4739/DLX3 axis on osteoporotic BMSC osteogenic differentiation **(A)** DLX3 knockdown was achieved in osteoporotic BMSCs by transfecting short hairpin RNA against DLX3 (sh1/2-DLX3); DLX3 knockdown was confirmed by qRT-PCR. n=3. Then, osteoporotic BMSCs were co-transfected with sh-DLX3 and antagomir-4739 and examined for the protein levels of DLX3 by Immunoblotting **(B)** and cell viability by CCK-8 assay **(C)** (n=3). Then, normal BMSCs were co-transfected with sh-DLX3 and antagomir-4739, induced towards osteogenic differentiation for 21 days, and examined using ALP staining on day 14 of osteogenic induction **(D)**; the formation of mineralized nodules using Alizarin red staining on day 21 of osteogenic induction **(E)**; the protein levels of ALP, OCN, Runx2, and Osterix using Immunoblotting on day 14 of osteogenic induction **(F)**. n=3. **P < 0.01, ^##^ *P <* 0.01.

After confirming miR-4739 binding to DLX3, the co-effects of miR-4739 and DLX3 upon the osteogenic differentiation capacity of BMSCs were evaluated. DLX3 knockdown was achieved in osteoporotic BMSCs through the transfection of short hairpin RNA against DLX3 (sh1/2-DLX3); DLX3 knockdown was confirmed by qRT-PCR (**Figure 6A**). Osteoporotic BMSCs were then co-transfected with sh-DLX3 and antagomir-4739 and examined for DLX3 protein levels. **Figure 6B** illustrated that miR-4739 inhibition increased, whereas DLX3 knockdown decreased DLX3 protein levels. The inhibitory effects of miR-4739 upon DLX3 protein levels were partially attenuated by DLX3 knockdown. Meanwhile, miR-4739 inhibition promoted, whereas DLX3 knockdown inhibited cell viability; DLX3 knockdown significantly attenuated the inhibitory effects of miR-4739 upon cell viability (**Figure 6C**).

Then, osteoporotic BMSCs were co-transfected with sh-DLX3 and antagomir-4739, induced towards osteogenic differentiation for 21 days, and examined for osteogenic differentiation. ALP staining revealed that, on day 14 of osteogenic induction, miR-4739 inhibition promoted, whereas DLX3 knockdown inhibited the osteoblast differentiation; the effects of miR-4739 inhibition were partially attenuated by DLX3 knockdown (**Figure 6D**). Alizarin red staining showed that, on day 21 of osteogenic induction, miR-4739 inhibition promoted, whereas DLX3 knockdown inhibited the formation of mineralized nodules; the effects of miR-4739 inhibition were partially attenuated by DLX3 knockdown (**Figure 6E**). Consistently, on day 14 of osteogenic induction, miR-4739 inhibition increased, whereas DLX3 knockdown decreased ALP, OCN, Runx2, and osterix protein contents. The effects of miR-4739 inhibition were partially attenuated by DLX3 knockdown (**Figure 6F**). These data indicate that miR-4739 influences BMSC osteogenesis through targeting DLX3.

## Discussion

Herein, the study identified that miR-4739 was upregulated in BMSC cultures established from osteoporotic subjects. BMSCs were isolated from normal and osteoporotic bone marrow tissues and identified for their osteogenic differentiation potential. In osteoporotic BMSCs, miR-4739 overexpression significantly inhibited cell viability, osteoblast differentiation, mineralized nodule formation and heterotopic bone formation, whereas miR-4739 inhibition exerted opposite effects. Through direct binding, miR-4739 inhibited DLX3 expression. In osteoporotic BMSCs, DLX3 knockdown also inhibited BMSC viability and osteogenic differentiation. DLX3 knockdown partially attenuated the effects of miR-4739 inhibition upon BMSCs.

Studies have revealed that the imbalance of BMSCs is an essential mechanism in the pathogenesis of osteoporosis (27, 28). The osteoblast-mediated bone formation is crucial for maintaining bone homeostasis (29, 30). In osteoporosis patients, BMSCs differentiate into more adipocytes than osteoblasts, resulting in continuous bone loss and accumulation of bone marrow fat (31, 32). Compared with normal BMSCs, osteoporotic BMSCs showed impaired osteoblast differentiation and mineralized nodule formation abilities, consistent with previous studies. Nevertheless, the mechanism underlying this shift of lineage commitment of BMSCs requires further investigation.

miRNAs were found to contribute extensively to regulating gene expression during life activities. Some miRNAs can promote osteogenic differentiation of BMSCs, while others exert opposite functions (33–35). miR-31 regulates osteogenic differentiation by targeting Runx2 and Satb2 formation regulatory loops (36). miR-204 affects the precursor by regulating Runx2 expression (37). miR-205 regulates BMSC osteogenic differentiation by influencing SATB2/Runx2 and ERK/MAPK pathways (38). Herein, it was identified that miR-4739 was upregulated within osteoporotic tissue samples and BMSCs. Conversely, miR-4739 expression was downregulated in BMSCs during osteogenic differentiation. As previously reported, miR-4739 could regulate the capacity of human BMSCs to differentiate into osteoblasts. Elsafadi et al. (39) demonstrated that hsa-miR-4739-transfected BMSCs exhibited impaired osteoblast differentiation. This was demonstrated by a significant reduction in the mineralized matrix formation, mineralized nodule quantification, and decreased expression of osteoblastic markers. Similarly, in this study, miR-4739 overexpression in normal and osteoporotic BMSCs inhibited mineralized matrix formation, suppressed the formation of mineralized nodules, and decreased the levels of osteoblastic markers, such as ALP, OCN, Runx2, and Osterix. Thus, our findings further confirm the inhibitory effects of miR-4739 upon the capacity of BMSCs to differentiate into osteoblasts.

miRNAs exert their functions through downregulating gene expression *via* translational repression or degradation of mRNA targets (8, 9). Elsafadi et al. (39) reported that miR-4739 modulates the capacity of immortalized human BMSCs to differentiate into osteoblasts and adipocytes *via* targeting LRP3. miR-4739 targeted regulation of Notch2 expression also regulated osteogenic differentiation (40). The multiple-to-multiple relations between miRNAs and mRNAs lead to the even more complex miRNA regulatory mechanisms (41). Herein, DLX3 was identified as a novel direct downstream target of miR-4739. DLX3, a homeodomain transcription factor, belongs to the DLX family that comprises 6 different members, DLX1-DLX6 (42, 43). DLX3 exerts a substantial effect on embryogenesis and organ development, such as epidermis and ectodermal appendages (44, 45). DLX3 promoted osteogenic differentiation of BMSCs and the induced pluripotent stem cell-derived mesenchymal stem cells (46, 47). DLX3 overexpression promoted the differentiation of BMSCs into osteoblasts through the Wnt/beta-catenin signaling-mediated histone methylation of DKK4 (26). Besides, DLX3 could inhibit adipogenic differentiation of dental pulp stem cells (48). Increased adipogenic differentiation or reduced osteogenic differentiation of BMSCs might lead to osteoporosis (49). Altogether, DLX3 may play a key role in osteoporosis. Herein, DLX3 knockdown in osteoporotic BMSCs leads to impaired mineralized matrix formation, mineralized nodule formation, and decreased levels of osteogenic markers, suggesting that DLX3 knockdown hindered BMSC osteogenic differentiation. More importantly, DLX3 knockdown partially reversed the inhibitory effects of miR-4739 upon BMSCs, suggesting that miR-4739 plays its role through DLX3 during the osteogenic differentiation. However, the function of miR-4739/DLX3 axis in adipogenic differentiation remains to be further studied.

Altogether, the miR-4739/DLX3 axis modulates the capacity of BMSCs to differentiate into osteoblasts, which are potentially involved in osteoporosis pathogenesis. The *in vivo* and clinical functions of the miR-4739/DLX3 axis require further investigation.

## Data Availability Statement

The original contributions presented in the study are included in the article/**Supplementary Material**. Further inquiries can be directed to the corresponding author.

## Ethics Statement

The studies involving human participants were reviewed and approved by Ethics Committee of the Second Xiangya Hospital of Central South University. The patients/participants provided their written informed consent to participate in this study. The animal study was reviewed and approved by Animal Care and Use Ethics Committee of Central South University.

## Author Contributions

DL: Writing- Reviewing and Editing, Project administration. QY: Writing- Reviewing and Editing. LX: Conceptualization, Methodology. AL: Data curation, Visualization, Investigation. YX: Software, Validation. All authors contributed to the article and approved the submitted version.

## Conflict of Interest

The authors declare that the research was conducted in the absence of any commercial or financial relationships that could be construed as a potential conflict of interest.

## Publisher’s Note

All claims expressed in this article are solely those of the authors and do not necessarily represent those of their affiliated organizations, or those of the publisher, the editors and the reviewers. Any product that may be evaluated in this article, or claim that may be made by its manufacturer, is not guaranteed or endorsed by the publisher.

## Supplementary Material

Supplementary Figure 1 Differentially expressed miRNAs in non-osteoporotic or osteoporotic tissues based on the GSE datasets. **(A)** In GSE74209, 83 upregulated miRNAs and 60 downregulated miRNAs in postmenopausal women bone tissues from osteoporotic fracture induced hip replacement compared to bone tissues from osteoarthritis without osteoporosis induced hip replacement based on (∣logFC∣ >1, p < 0.05). The top 20 differentially expressed miRNAs were shown in the heatmap. **(B)** In GSE93883, 20 upregulated miRNAs and 117 downregulated miRNAs in plasma from osteoporotic patients with and without vertebral fractures compared to non-osteoporotic patients(|logFC| >1, p < 0.05). The top 20 differentially expressed miRNAs were shown in the heatmap.

Supplementary Figure 2 Effects of miR-4739 overexpression and inhibition on normal BMSC osteogenic differentiation **(A)** miR-4739 overexpression or inhibition was achieved in normal BMSCs by transfecting agomir-4739 or antagomir-4739; miR-4739 expression was confirmed using qRT-PCR. n=3. **(B)** Normal BMSCs were transfected with agomir-4739 or antagomir-4739 and examined for cell viability by CCK-8 assay. n=3. Then, normal BMSCs were transfected with agomir-4739 or antagomir-4739, induced towards osteogenic differentiation for 21 days, and examined using ALP staining on day 14 of osteogenic induction **(C)**; examined for the formation of mineralized nodules using Alizarin red staining on day 21 of osteogenic induction **(D)**; examined for the protein levels of ALP, OCN, Runx2, and Osterix using Immunoblotting on day 14 of osteogenic induction **(E)**. n=3. ***P <*0.01, compared to NC agomir; ^#^ *P <*0.05, ^##^ *P <*0.01. compared to NC antagomir.
