# Supplementary figures and images for: The miR-4739/DLX3 Axis Modulates Bone Marrow-Derived Mesenchymal Stem Cell (BMSC) Osteogenesis Affecting Osteoporosis Progression
Source: Front Endocrinol (Lausanne). 2021 Dec 2;12:703167. doi: 10.3389/fendo.2021.703167 (PMC8678599; doi:10.3389/fendo.2021.703167)

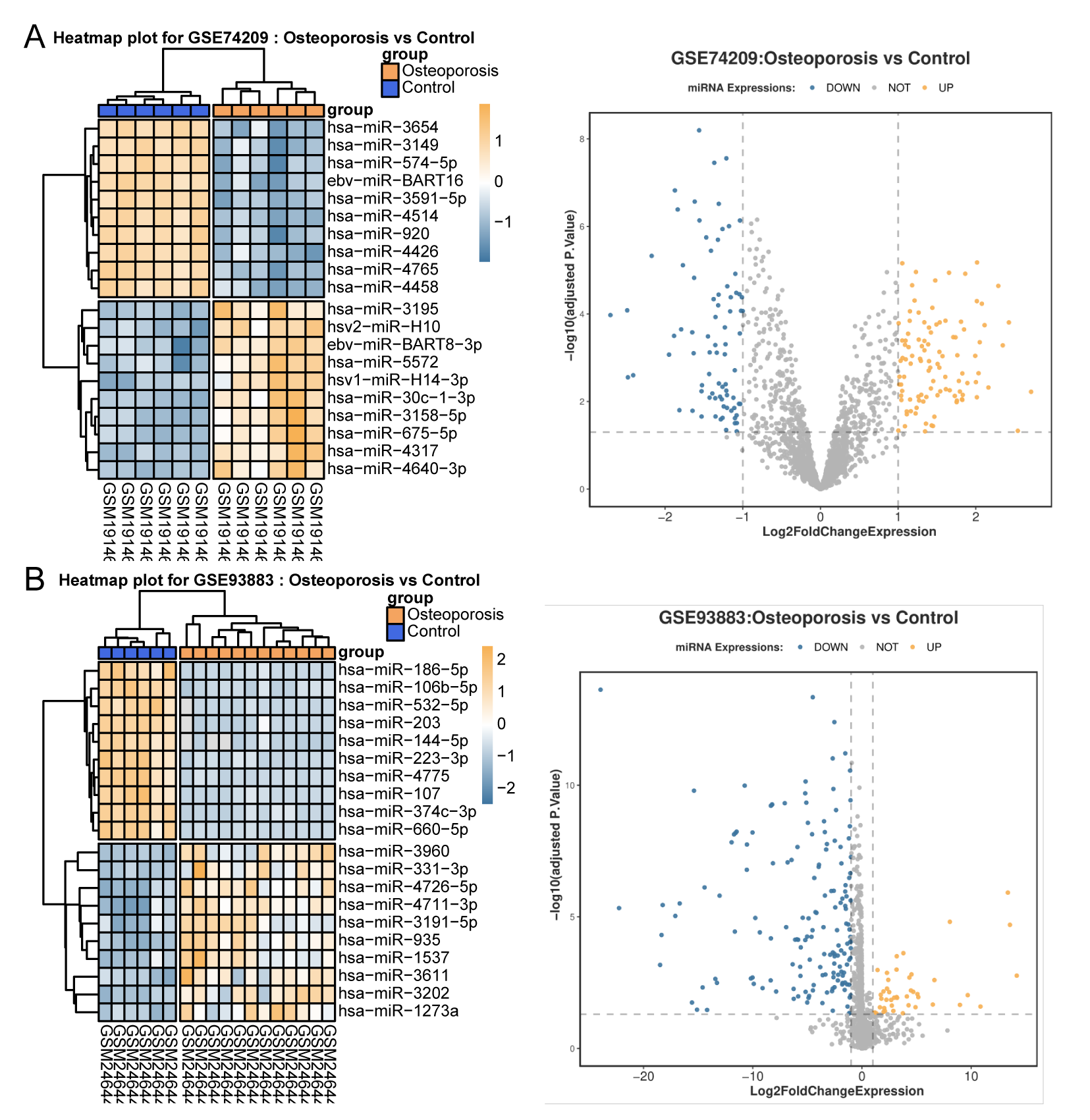

Supplement: Supplementary Figure 1 — Differentially expressed miRNAs in non-osteoporotic or osteoporotic tissues based on the GSE datasets. (A) In GSE74209, 83 upregulated miRNAs and 60 downregulated miRNAs in postmenopausal women bone tissues from osteoporotic fracture induced hip replacement compared to bone tissues from osteoarthritis without osteoporosis induced hip replacement based on (∣logFC∣ >1, p < 0.05). The top 20 differentially expressed miRNAs were shown in the heatmap. (B) In GSE93883, 20 upregulated miRNAs and 117 downregulated miRNAs in plasma from osteoporotic patients with and without vertebral fractures compared to non-osteoporotic patients(|logFC| >1, p < 0.05). The top 20 differentially expressed miRNAs were shown in the heatmap. [file Image_1.tif]

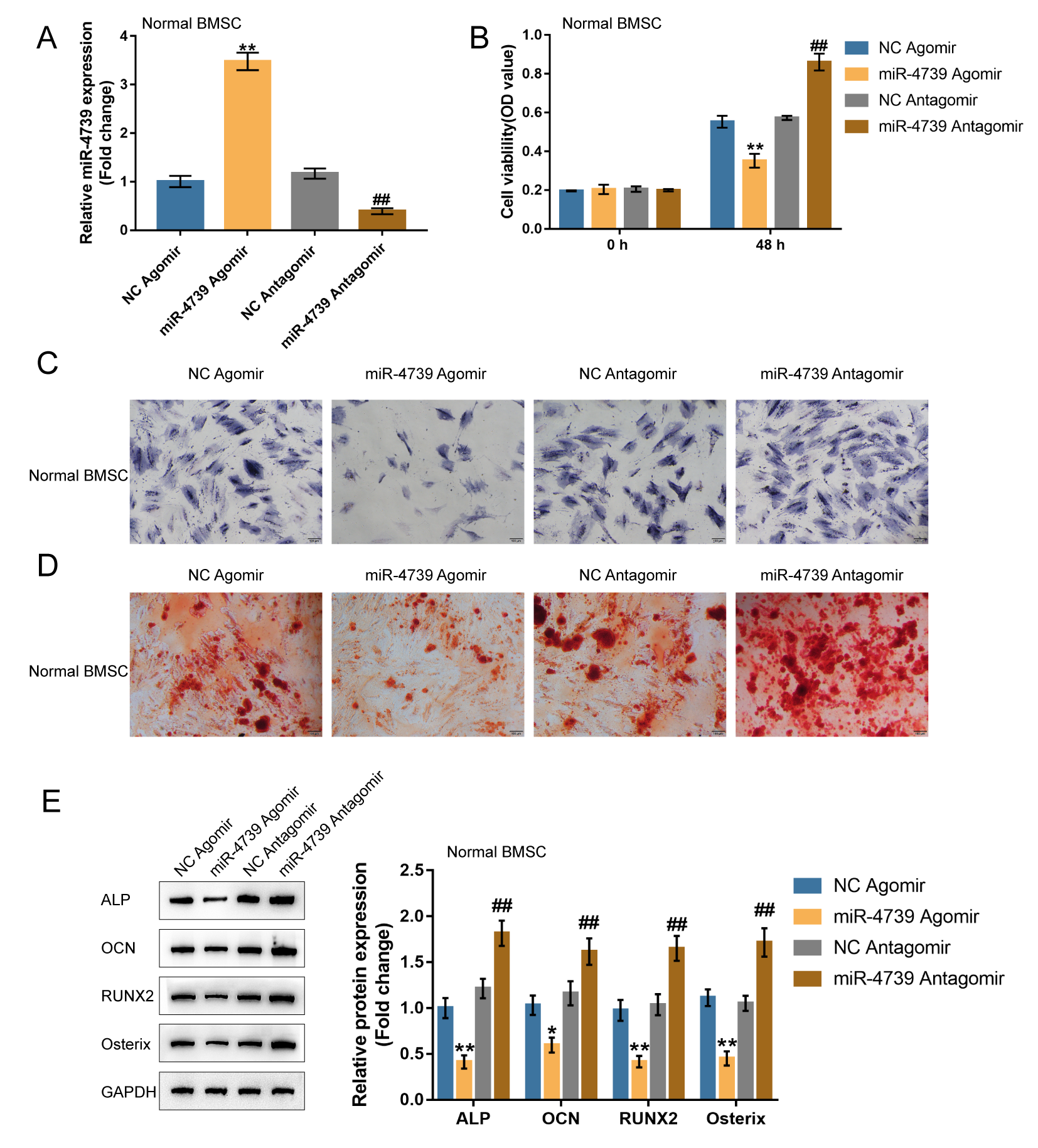

Supplement: Supplementary Figure 2 — Effects of miR-4739 overexpression and inhibition on normal BMSC osteogenic differentiation (A) miR-4739 overexpression or inhibition was achieved in normal BMSCs by transfecting agomir-4739 or antagomir-4739; miR-4739 expression was confirmed using qRT-PCR. n=3. (B) Normal BMSCs were transfected with agomir-4739 or antagomir-4739 and examined for cell viability by CCK-8 assay. n=3. Then, normal BMSCs were transfected with agomir-4739 or antagomir-4739, induced towards osteogenic differentiation for 21 days, and examined using ALP staining on day 14 of osteogenic induction (C); examined for the formation of mineralized nodules using Alizarin red staining on day 21 of osteogenic induction (D); examined for the protein levels of ALP, OCN, Runx2, and Osterix using Immunoblotting on day 14 of osteogenic induction (E). n=3. **P <0.01, compared to NC agomir; # P <0.05, ## P <0.01. compared to NC antagomir. [file Image_2.tif]
