# Supplementary material for: The miR-4739/DLX3 Axis Modulates Bone Marrow-Derived Mesenchymal Stem Cell (BMSC) Osteogenesis Affecting Osteoporosis Progression
Source: Front Endocrinol (Lausanne). 2021 Dec 2;12:703167. doi: 10.3389/fendo.2021.703167 (PMC8678599; doi:10.3389/fendo.2021.703167)
Supplement: Supplementary file 3 [file Table_1.docx]

Table S1 the primer sequence

| RT-PCR  MiR-4739 | Rt: GTCGTATCCAGTGCGTGTCGTGGAGTCGGCAATTGCACTGGATACGACAGGGCC  AAGGGAGGAGGAGCGGAGG | CAGTGCGTGTCGTGGA |
| --- | --- | --- |
| RT-PCR  MiR-3202 | GTCGTATCCAGTGCGTGTCGTGGAGTCGGCAATTGCACTGGATACGACATTAAA  GCTGGAAGGGAGAAGAGC | CAGTGCGTGTCGTGGA |
| RT-PCR  MiR-320c | GTCGTATCCAGTGCGTGTCGTGGAGTCGGCAATTGCACTGGATACGACACCCTC  GCCAAAAGCTGGGTTGA | CAGTGCGTGTCGTGGA |
| RT-PCR  U6 | CTCGCTTCGGCAGCACA | AACGCTTCACGAATTTGCGT |
| RT-PCR  DLX3 | TACCCTGCCCGAGTCTTCTG | TGGTGGTAGGTGTAGGGGTTC |
| RT-PCR  LASP1 | CGAGAAGAAGCCCTACTGCAA | CTGCCACTACGCTGAAACCT |
| RT-PCR  GAPDH | ACAGCCTCAAGATCATCAGC | GGTCATGAGTCCTTCCACGAT |
| NC agomir | UUCUCCGAACGUGUCACGUTT | ACGUGACACGUUCGGAGAATT |
| NC antagomir | CAGUACUUUUGUGUAGUACAA |  |
| MiR-4739 agomir | AAGGGAGGAGGAGCGGAGGGGCCCU | GGCCCCUCCGCUCCUCCUCCCUUUU |
| MiR-4739 antagomir | AGGGCCCCUCCGCUCCUCCUCCCUU |  |
| Wt-DLX3 3’UTR | aattctaggcgatcgctcgagACCCAGACATCCCACCAAAGC | attttattgcggccagcggccgcACTCTTGGAGACCTACCACGTCTC |
| Mut-DLX3 3’UTR | AAGCAGCCTcacggatGCTGCGGTTTCCTATTTATGTGG | CatccgtgAGGCTGCTTCTCTCTGTTGCTCTT |
| Sh-NC | GATCCACACAGCAGGTCAAGAGGAGTCTCGAGACTCCTCTTGACCTGCTGTGTTTTTTG | AATTCAAAAAACACAGCAGGTCAAGAGGAGTCTCGAGACTCCTCTTGACCTGCTGTGTG |
| Sh1-DLX3 | GATCCGCTGGAGCACAGTCCCAATAACTCGAGTTATTGGGACTGTGCTCCAGCTTTTTG | AATTCAAAAAGCTGGAGCACAGTCCCAATAACTCGAGTTATTGGGACTGTGCTCCAGCG |
| Sh2-DLX3 | GATCCGCACTTCTAGCCCTCATTTAACTCGAGTTAAATGAGGGCTAGAAGTGC TTTTTG | AATTCAAAAAGCACTTCTAGCCCTCATTTAACTCGAGTTAAATGAGGGCTAGAAGTGCG |
